# A *Dhdds* K42E knock-in RP59 mouse model shows inner retina pathology and defective synaptic transmission

**DOI:** 10.1038/s41419-023-05936-4

**Published:** 2023-07-13

**Authors:** Mai N. Nguyen, Dibyendu Chakraborty, Sriganesh Ramachandra Rao, Agnieszka Onysk, Mariusz Radkiewicz, Liliana Surmacz, Ewa Swiezewska, Eric Soubeyrand, Tariq A. Akhtar, Timothy W. Kraft, David M. Sherry, Steven J. Fliesler, Steven J. Pittler

**Affiliations:** 1 Department of Optometry and Vision Science, Vision Science Research Center, School of Optometry, University of Alabama at Birmingham, Birmingham, AL 35294 USA; 2 Research Service, VA Western New York Healthcare System, Buffalo, NY 14215 USA; 3 Departments of Ophthalmology and Biochemistry and Neuroscience Graduate Program, Jacobs School of Medicine and Biomedical Sciences, The State University of New York, University at Buffalo, Buffalo, NY 14203 USA; 4 Institute of Biochemistry and Biophysics, Polish Academy of Sciences, Warsaw, 02106 Poland; 5 Department of Molecular and Cellular Biology, University of Guelph, Guelph, Ontario, N1G2W1 Canada; 6 Departments of Cell Biology, Neurosurgery, and Pharmaceutical Sciences, University of Oklahoma Health Sciences Center, Oklahoma City, OK 73104 USA

**Keywords:** Glycoproteins, Glycolipids, Neurophysiology, Ion channel signalling

## Abstract

Retinitis pigmentosa (RP) defines a group of hereditary progressive rod-cone degenerations that exhibit a common phenotype caused by variants in over 70 genes. While most variants in the dehydrodolichyl diphosphate synthase (DHDDS) gene result in syndromic abnormalities, some variants cause non-syndromic RP (RP59). DHDDS encodes one subunit of the enzyme *cis*-prenyltransferase (CPT), which is required for the synthesis of dolichol (Dol), that is a necessary protein glycosylation cofactor. We previously reported the creation and initial characterization of a knock-in (KI) mouse model harboring the most prevalent RP59-associated *DHDDS* variant (K42E) to understand how defects in DHDDS lead to retina-specific pathology. This model exhibited no profound retinal degeneration, nor protein *N*-glycosylation defects. Here, we report that the Dol isoprenylogue species in retina, liver, and brain of the K42E mouse model are statistically shorter than in the corresponding tissues of age-matched controls, as reported in blood and urine of RP59 patients. Retinal transcriptome analysis demonstrated elevation of many genes encoding proteins involved in synaptogenesis and synaptic function. Quantitative retinal cell layer thickness measurements demonstrated a significant reduction in the inner nuclear layer (INL) and total retinal thickness (TRT) beginning at postnatal (PN) ∼2 months, progressively increasing to PN 18-mo. Histological analysis revealed cell loss in the INL, outer plexiform layer (OPL) disruption, and ectopic localization of outer nuclear layer (ONL) nuclei into the OPL of K42E mutant retinas, relative to controls. Electroretinograms (ERGs) of mutant mice exhibited reduced b-wave amplitudes beginning at PN 1-mo, progressively declining through PN 18-mo, without appreciable a-wave attenuation, relative to controls. Our results suggest that the underlying cause of *DHDDS* K42E variant driven RP59 retinal pathology is defective synaptic transmission from outer to inner retina.

## Introduction

Retinitis pigmentosa (RP) is comprised of a genetically diverse family of hereditary retinal degenerative diseases that share several features, such as progressive retinal degeneration, diminished or absent electroretinographic (ERG) responses to light stimulation, bone-spicule like pigmentary changes, a waxy pallor of the optic disk, and varying degrees of macular involvement [1–6]. One form of RP (RP59, OMIM#613861) involves variants in the gene that encodes dehydrodolichyldiphosphate synthase (DHDDS), which together with Nogo-B receptor (NgBR) forms the *cis-*prenyltransferase (CPT) enzyme complex [7]. CPT catalyzes isoprenoid chain elongation, by stepwise addition of 5-carbon units (“isoprene”), using isopentenyl diphosphate (IPP) as a co-substrate to farnesyl diphosphate (FPP), generating polyprenyl diphosphate, to species typically containing 95 to 110 carbons in mammals. Dephosphorylation and reduction produces dolichyldiphosphate/dolichol (Dol-PP/Dol). Dolichol is required for protein *N*-glycosylation and several other forms of glycosylation [8–10]. In plasma and urine from RP59 patients, Dol chain lengths were shorter than normal [11]; Dol containing 19 isoprene units (Dol-19) is the predominant species in human bodily tissues and fluids; however, in RP59 patients, there is a shift to Dol species containing 18 and 17 isoprene units (Dol-18 and Dol-17). In fact, the Dol-18/Dol-19 ratio was diagnostic of RP59 and could be distinguished between human subjects harboring homozygous vs. heterozygous *DHDDS* variants and normal patients [11].

The first described cases of *DHDDS*-associated, non-syndromic autosomal recessive retinitis pigmentosa RP59 [12, 13] involved a founder missense variant *DHDDS*^K42E/K42E^ which alters the CPT catalytic domain [14, 15]. An RP screen within an Ashkenazi Jewish population suggested that 33% of screened RP cases were associated with the *DHDDS*^K42E/K42E^ point variant [16]. Other DHDDS variants have been reported in RP59 patients (e.g., T206A, R98W; both identified as compound heterozygous with K42E) [11, 16]. A patient with severe bradycardia and arrested physical growth with a *DHDDS* W64X and a splice variant, exhibited a null ERG at 2 mo and died at 8 mo of age [17]. Surprisingly, several *DHDDS* dominant variants around the active site led to progressive encephalopathy and myoclonus, without involvement of retinal dystrophy [18, 19]. A key clinical diagnostic feature of K42E-associated RP59 is shortening of Dol species (in patient urine or serum) [11], while encephalopathy-associated variants do not lead to altered Dol metabolism [19]. Neither class of *DHDDS* variants exhibit a serum transferrin glycosylation defect that is the hallmark of many forms of congenital disorder of glycosylation (CDG) [20].

To understand the underlying mechanism of RP59, and the potential role of abnormalities of Dol synthesis in retinal dysfunction and degeneration, we generated and partially characterized a *Dhdds*^K42E/K42E^ knock-in (herein referred to as K42E) mouse model [21] of human K42E RP59. Retinas of K42E mice exhibited grossly normal histology in comparison to age-matched wildtype (WT) mice over a postnatal (PN) 1-mo to 12-mo time course. However, substantial and persistent gliotic reactivity was observed in K42E retinas. Global retinal protein *N*-glycosylation, however, was not altered [21].

Here, we demonstrate that the K42E mouse line exhibits key biochemical and diagnostic features of RP59. Further, we show structural and inner retina functional deficits in the K42E mouse model consistent with altered synaptic transmission between retinal photoreceptors and bipolar cells. This investigation may provide novel insights into RP59 disease mechanism that will guide future testing of therapeutic interventions to slow or halt the progression of this blinding disorder.

## Results

### The Dol-17/Dol-18 ratio in retina, liver, and brain is greater than WT in K42E *Dhdds* mutant mice

**Fig. 1:**
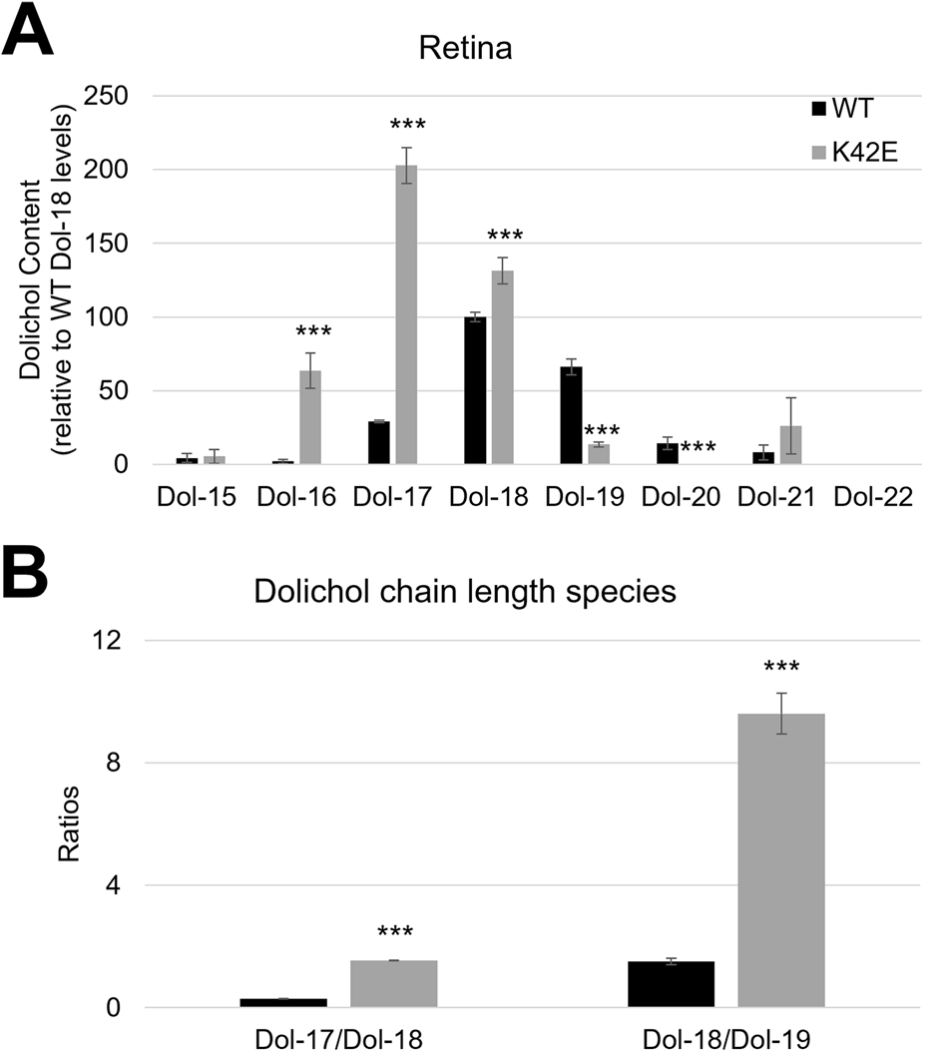
Dol chain length percentages (relative to WT Dol-18) and Dol ratios in WT and KI retinas. Percentages of Dol chain lengths of WT (*black*) and K42E (*gray*) retina relative to WT Dol-18 levels are shown in (**A**). Dol-16, Dol-17, and Dol-18 levels are significantly increased in K42E retinas compared to WT. Dol-17/Dol-18 and Dol-18/Dol-19 ratios (**B**) are both significantly higher than WT. Statistical significance: ***p ≤ 0.001. K42E, *n* = 4; WT, *n* = 5.

An increase in Dol-18/Dol-19 ratio has been reported to be diagnostic for RP59 and was used to distinguish between homozygous human subjects and normal subjects [11]. In normal human subjects, blood and urine analyses showed that Dol-19 is the dominant Dol species, whereas in RP59 patients harboring either two K42E mutant alleles or one K42E and one T206A mutant alleles, the dominant isoprenylogue is Dol-18, due to shortening of Dol chain length [11, 14, 15]. Here, we performed UPLC-MS analysis of tissue Dol profile from age-matched WT and K42E knock-in mice. We compared Dol chain length levels relative to WT Dol-18 (Fig. 1A) as well as ratios of Dol-17/Dol-18 and Dol-18/Dol-19 (Fig. 1B) in WT and K42E mouse retina (see Suppl. Table 1). In KI mice, Dol-17 and Dol-18 levels expressed as a percentage of WT Dol-18 levels were greatly increased, while Dol-18 and Dol-19 were highest in WT retinas. In addition, Dol-17/Dol-18 and Dol-18/Dol-19 ratios were increased in K42E retinas compared to WT. Furthermore, we compared Dol species in brain and liver tissues (Suppl. Fig. 1 A–D and Suppl. Table 1) finding that the predominant species in WT mouse brain and liver were Dol-18 and Dol-19, whereas in the K42E retina, Dol-16 and Dol-17 were increased and Dol-18 was either unchanged (liver) or greatly increased (brain), relative to WT levels. There were major reductions in Dol-19 and Dol-20 in mutant tissues (less pronounced for Dol-19 in liver). Hence, the K42E variant *does not* inhibit the synthesis of Dol (i.e., there is no evidence to support classifying the variant as a loss-of-(DHDDS) function variant), but rather it leads to shortening of Dol chain length.

### Transcriptome profiling identifies altered transcript levels in many genes involved in synaptogenesis

We adapted an unbiased, omic-approach to reveal potential pathways affected by *Dhdds* K42E variant. We assessed transcriptomic changes in retinas from K42E *vs*. age-matched WT mice using NanoString® RNASeq analysis. Sixty-eight differentially regulated genes were identified in K42E mouse retina, with 54 exhibiting up- and 14 exhibiting down-regulation (Suppl. Table 2). Analysis of the results revealed differential transcription of many genes involved in synaptogenesis and synaptic function. These results prompted a closer inspection of the structure and function of the inner and outer retina.

### The K42E *Dhdds* variant affects retinal structure

**Fig. 2:**
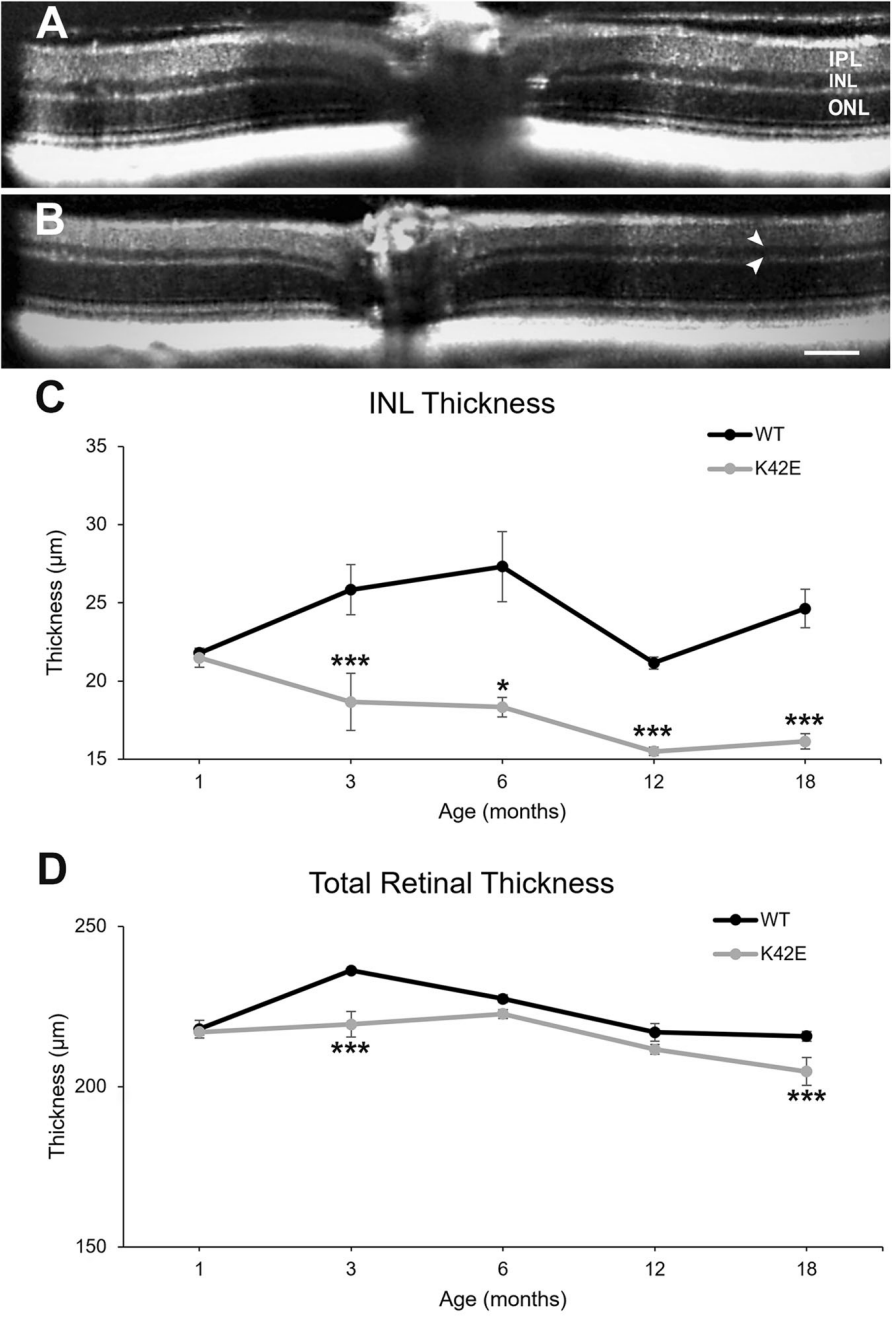
INL and total retinal thickness is significantly reduced in retinas of K42E vs. WT mice. Representative SD-OCT images from PN 18-mo old WT (**A**) and K42E (**B**) SD-OCT images are shown. White arrowheads note the thinning of the K42E retina INL. Averaged INL (**C**) and total retinal (**D**) thickness from PN 1- to 18-mo shows progressive reduction of INL thickness in K42E mice (*gray*) compared to WT mice (*black*) and significantly lower total retinal thickness at PN 3-mo and 18-mo. IPL inner plexiform layer, INL inner nuclear layer, ONL outer nuclear layer. Scale bar: 90 µm, panels **A**, **B**. Statistical significance: *p ≤ 0.05, ***p ≤ 0.001. K42E PN 1-mo, *n* = 3, PN 3-mo, *n* = 3, PN 6-mo, *n* = 3, PN 12-mo, *n* = 6, PN 18-mo, *n* = 3; WT PN 1-mo, *n* = 4, PN 3-mo, *n* = 4, PN 6-mo, *n* = 5, PN 12-mo, *n* = 9, PN 18-mo, *n* = 4.

Previously, we reported that spectral domain optical coherence tomography (SD-OCT) measurements of total retinal and outer nuclear layer (ONL) thicknesses in K42E mice were comparable to age-matched WT retinas at each time point examined from PN 1-mo to PN 12-mo [21]. Based on the transcriptome analysis, we refocused our structural analysis on comparison of the inner retina in mice from ages PN 1- to 18-mo. At 18-mo, SD-OCT analysis of K42E and WT retina showed significant reduction in inner nuclear layer (INL) thickness in K42E mice (see arrows in Fig. 2B), while stratification of all cell layers appeared comparable to those of age-matched WT mice (Fig. 2A). We observed a progressive reduction in INL thickness with increasing age (Fig. 2C, D, 34.5%,), at PN 3- (*p* ≤ 0.001), 6- (*p* ≤ 0.05), 12- (*p* ≤ 0.001), and 18-mo (*p* ≤ 0.001). We also re-evaluated TRT in larger numbers of K42E and WT mice, and found a small, but significant reduction at PN 3- and 18-mo (Fig. 2D, *p* ≤ 0.001) compared to WT. The lack of a difference between normal and mutant mice comparing INL and TRT measurements at PN 1-mo is consistent with the effects of the variant being post-developmental (i.e., ensuing only after differentiation of all retinal cell types and completion of histological stratification).

**Fig. 3:**
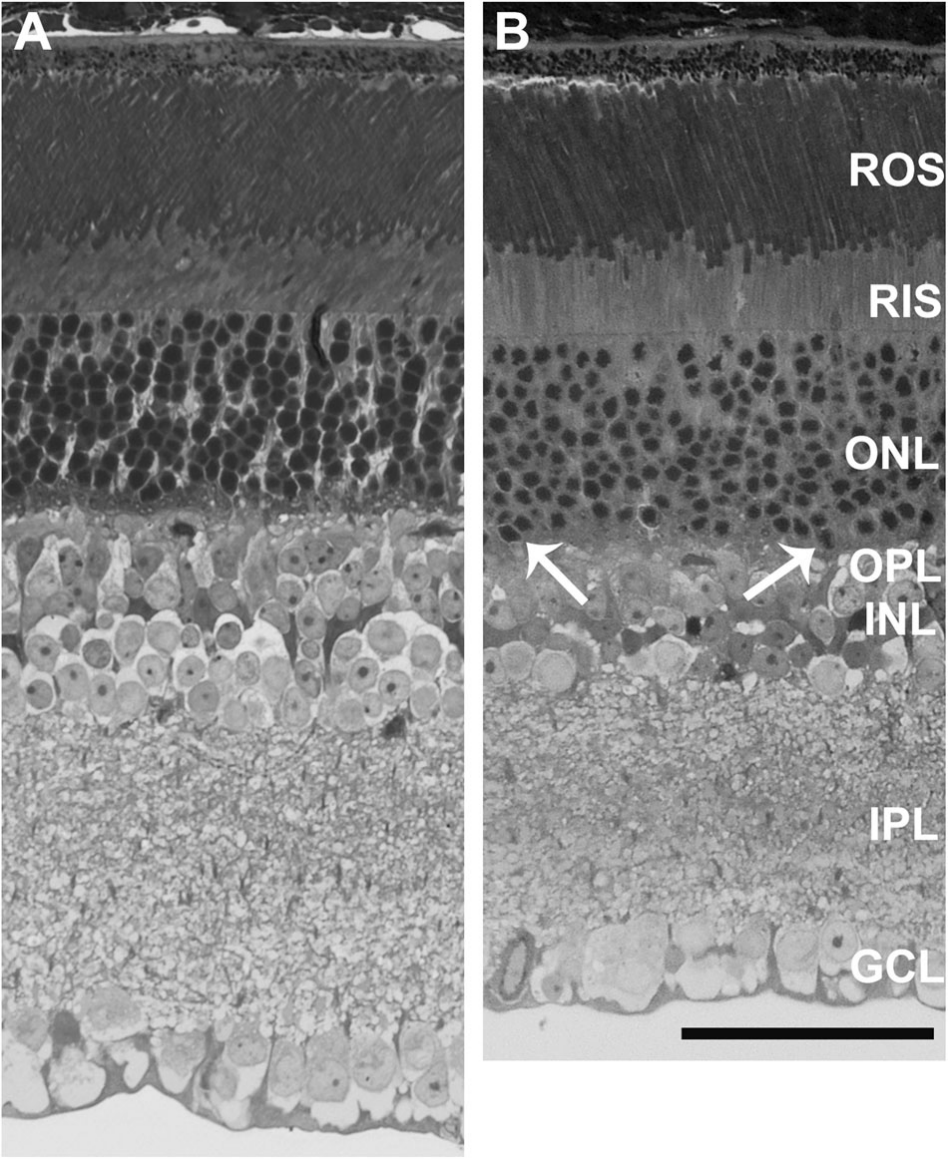
Comparative histology of K42E and WT retinas demonstrate retinal thinning and nuclear migration in the K42E retina. WT (**A**) and K42E (**B**) retinal micrographs show reduced TRT and alteration of the inner retina in K42E. Migration of ONL nuclei is apparent in the K42E retina (*arrows*). Reduced thickness in the K42E OPL, INL, and IPL compared to WT is indicative of cell loss. ROS rod outer segments, RIS rod inner segments, ONL outer nuclear layer, OPL outer plexiform layer, INL inner nuclear layer, IPL inner plexiform layer, GCL ganglion cell layer. Scale bar: 50 µm, both panels.

To study changes in gross morphology, we performed morphological analysis of neural retina. Toluidine blue-staining of K42E retina (Fig. 3B) showed reduction in TRT compared to the WT retina (Fig. 3A). Specifically, thickness of the INL in K42E retina was visibly reduced compared to WT, consistent with SD-OCT findings. In contrast, ONL thickness did not differ between the K42E and WT retina. However, photoreceptor nuclei infiltrating the outer plexiform layer (OPL) were apparent in the K42E retina, suggesting pathology (Fig. 3B, arrows).

**Fig. 4:**
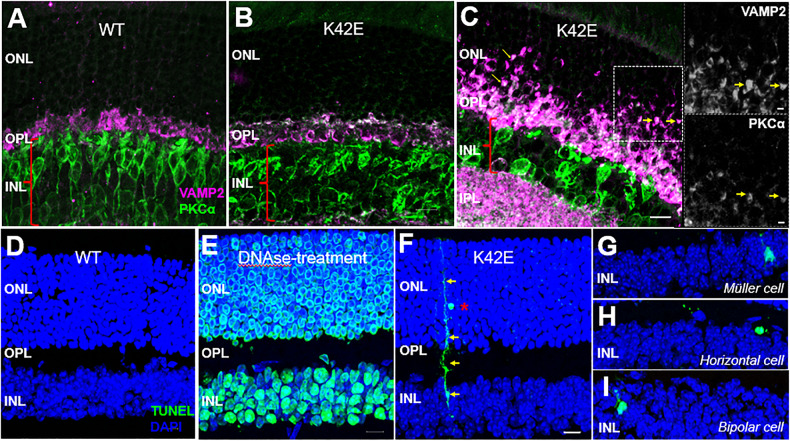
Structure of inner retina is altered in K42E relative to WT mice. Immunofluorescence analyses were performed on WT and K42E frozen retinal sections. Co-labeling of anti-VAMP2 (magenta)/ PKC-α (green) of WT (PN 19-mo) (**A**), K42E (PN 1-mo) (**B**), and K42E (PN 6-mo) (**C**) show early signs of dendritic retraction in the ONL, indicated by yellow arrows. Insets show co-labeling of VAMP2 (top) and PKCα (bottom). No TUNEL-positive staining (*green*) was observed in the WT retina **(**PN 6-mo, **D**) compared to the DNAse-treated retina (positive control, (**E**)). **F** TUNEL-positive staining (yellow arrows), indicating a photoreceptor (*red asterisk*) being phagocytosed by Müller glia in K42E retina at PN 1-mo. Further TUNEL staining indicated cell death of Muller cell (**G**), horizontal cell (**H**), and bipolar cell (**I**) in the INL. ONL outer nuclear layer, OPL outer plexiform layer, INL inner nuclear layer. Scale bar: 10 µm, all panels.

To further assess the K42E retina, we performed immunolabeling using cell- and synapse-specific markers (Table 1) to examine photoreceptor terminal retraction from the OPL, "sprouting" of processes from second-order neurons into the ONL, and synaptic ectopia in the inner retina [22, 23]. In aged WT retina, photoreceptor pre-synapses were restricted to the OPL and showed labeling for pre-synaptic vesicles (VAMP2) as expected. In contrast, the K42E retina showed pronounced retraction of photoreceptor terminals into the ONL by PN 6-mo (Fig. 4A–C). Retraction of photoreceptor terminals was accompanied by sprouting of rod bipolar dendrites (PKCα) into the ONL of the K42E retina (see Inset, Fig. 4C). Immunolabeling for GFAP, an indicator of Müller cell reactivity (“gliosis” [24]), was present at 6 and 12 mo in the K42E retina, but was absent from Müller cells in WT retinas at any age, as reported previously [21]. Analysis of other synaptic markers such as Synaptotagmin-1 (SYT1) confirmed photoreceptor terminal retraction in the K42E mouse model (Suppl. Fig. 2, arrows).

**Table 1:** Antibodies used in this work.

Antibody	Host	Dilution	Vendor	RRID
SYT-1	Rabbit, polyclonal	1:500	ABclonal, A0992	AB_2757511
VAMP2	Rabbit, monoclonal	1:200	CST, 13508	AB_2798240
PKCα	Mouse, monoclonal	1:100	ABclonal, ab11723	AB_298510

To assess cell death, we performed TUNEL staining in WT and K42E retina (Fig. 4D–I). The WT retina showed no TUNEL labeling (Fig. 4D, PN 6-mo). Pretreatment of tissue sections with DNase1 served as a TUNEL positive control (Fig. 4E). We observed slow cell death in all retinal layers, including photoreceptors, starting at PN 1-mo (Fig. 4F). Interestingly, Müller glial cells (yellow arrows, Fig. 4F) were involved in clearance of TUNEL-positive photoreceptor nuclei. In addition, TUNEL-positive Müller glia (Fig. 4G), horizontal cells (Fig. 4H), and bipolar cells (Fig. 4I) were also observed in the K42E retina as early as PN 1-mo, confirming low levels of cell loss in the INL of the K42E retina.

### Expression of K42E *Dhdds* leads to early decline of dark-adapted (DA) and light-adapted (LA) b-wave/a-wave amplitude ratio

**Fig. 5:**
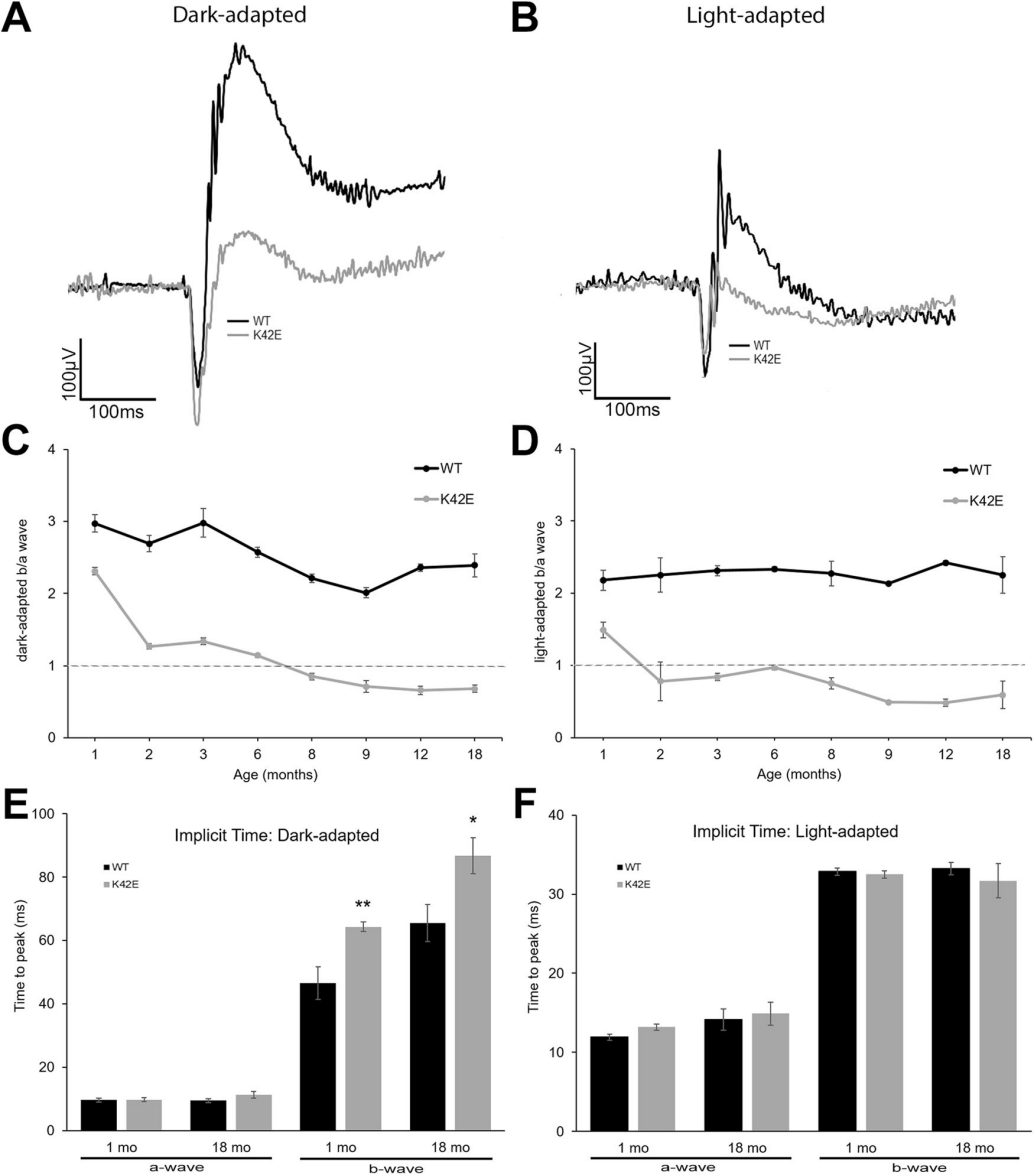
Attenuated b-wave amplitudes lead to reduced b/a ratios and slower responses in mutant mice. Representative traces of DA (**A**) and LA (**B**) ERG responses are shown for WT (black) *vs*. K42E mice (gray) at PN 6-mo. The DA b/a (**C**) and LA b/a (**D**) ERG responses were calculated and plotted over time for PN 1-, 2-, 3-, 6-, 8-, 9-, 12-, and 18-mo. All K42E b/a were significantly different from WT at all time points under both DA and LA conditions. Horizontal dashed line at b/a = 1 indicates the “negative ERG” threshold. Implicit time of a-waves and b-waves were averaged and plotted for DA (**E**) and LA (**F**) responses. Statistical significance: *p ≤ 0.05, **p ≤ 0.01. K42E PN 1-mo, *n* = 9, PN 2-mo, *n* = 11, PN 3-mo, *n* = 9, PN 6-mo, *n* = 15, PN 8-mo, *n* = 3, PN 9-mo, *n* = 3, PN 12-mo, *n* = 7, PN 18-mo, *n* = 10; WT PN 1-mo, *n* = 10, PN 2-mo, *n* = 7, PN 3-mo, *n* = 9, PN 6-mo, *n* = 14, PN 8-mo, *n* = 3, PN 9-mo, *n* = 5, PN 12-mo, *n* = 6, PN 18-mo, *n* = 8.

Little information is currently available on retinal function in RP59 patients. Lam et al. reported on three patients with low-to-undetectable ERG responses [25], while Kimchi et al. reported results from a cohort of patients with greatly reduced 30 Hz cone flicker amplitudes [26]. To evaluate function of the K42E retina, we performed full field ERGs (Fig. 5). Shown in Fig. 5A, B are representative dark-adapted (DA) and light-adapted (LA) ERG recordings obtained from PN 6-mo mice. We observed a significantly reduced b-wave amplitude (Suppl. Table 3) and DA b-wave/a-wave amplitude ratio (b/a ratio) in the K42E retina (22.2%, *p* ≤ 0.01) at PN 1-mo of age compared to WT (Fig. 5C). At PN 2-mo, there was a further b/a reduction (45%, *p* ≤ 0.001) relative to age-matched WT controls. At PN 8-mo, the DA b/a ratio dropped below 1, termed “negative ERG” [27], and declined further reduced with age. In contrast, WT b/a ratios were only marginally reduced over 18 mo (24%, *p* ≤ 0.05). Similarly, LA ERG analysis showed significantly reduced b/a ratio in the K42E retina at PN 1-mo (*p* ≤ 0.0001, Fig. 5D) *vs*. WT. By PN 2-mo, the LA K42E mice showed negative ERG while WT ratio changes were not significant month after month. The implicit time, or time-to-peak, was not significantly different for a-waves under either DA or LA conditions (Fig. 5E, F). However, K42E DA b-wave implicit time was 38% greater than WT at PN 1-mo and 32% greater than WT at PN 18-mo (*p* ≤ 0.05, Fig. 5E). However, the LA b-wave implicit times were not significantly different from WT (Fig. 5F).

### c-wave and d-wave analysis in WT and K42E retina

**Fig. 6:**
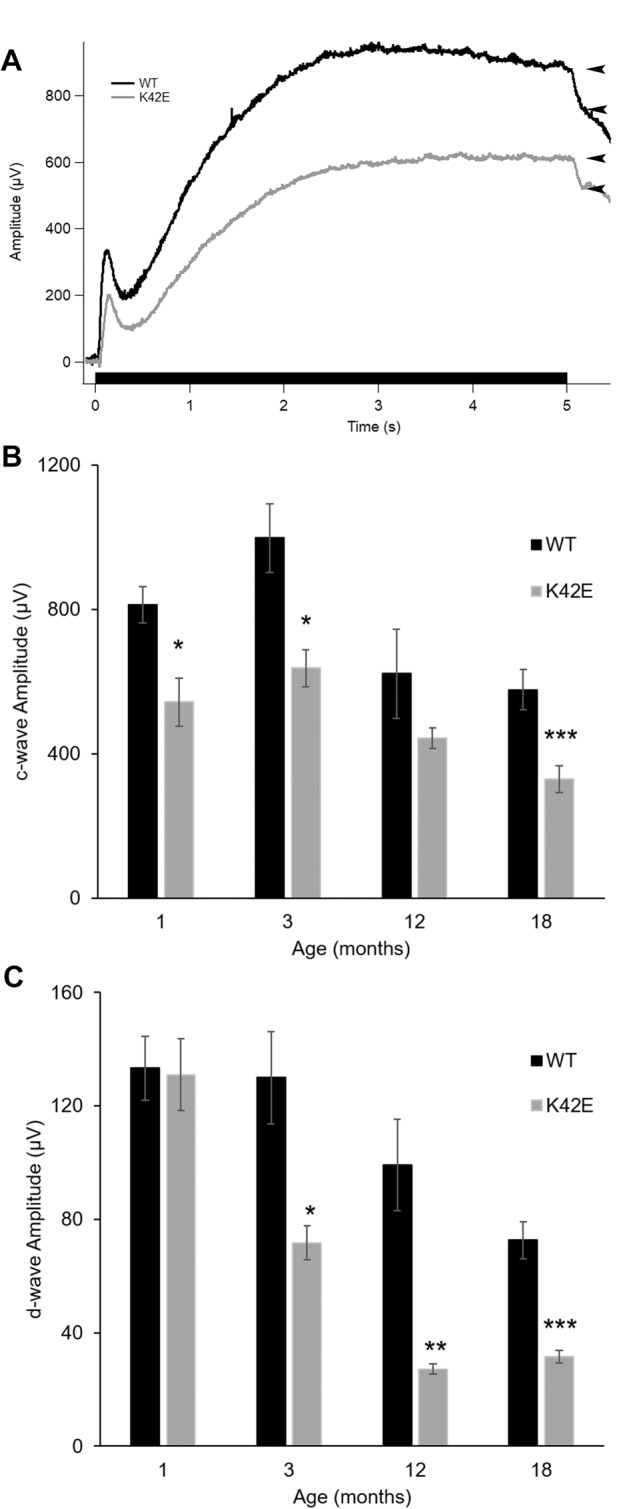
*Dhdds*^K42E/K42E^ mice exhibit RPE electrophysiological abnormality compared to WT mice. Representative c-wave responses are shown for WT (*black*) and K42E (gray) (**A**). Black arrowheads show the measurement points of the d-wave amplitudes. The c-wave (**B**) and d-wave (**C**) amplitudes were averaged and graphed for... and18-mo mice. Statistical significance: *p ≤ 0.05, **p ≤ 0.01, ***p ≤ 0.001. K42E PN 1-mo *n* = 9, PN 3-mo *n* = 9, PN 12-mo *n* = 6, PN 18-mo *n* = 8; WT PN 1-mo *n* = 9, PN 3-mo *n* = 5, PN 12-mo *n* = 7, PN 18-mo *n* = 10.

Previously, we observed that targeted ablation of *Dhdds* in the RPE caused RPE atrophy, retinal degeneration, and reduction of DA and LA ERG [28]. Here, we investigated the effect of the KI K42E *Dhdds* variant on RPE function by examining the c-wave, which reflects RPE and Müller cell contributions [29]. Figure 6A shows representative traces of DC ERG responses obtained from WT and K42E mice. As seen in Fig. 6B, there was a significant reduction of c-wave amplitude at PN 1-mo (*p* ≤ 0.05) and 3-mo (*p* ≤ 0.05). At PN 18-mo, there was further decline in c-wave amplitude (by 42.9%, *p* ≤ 0.001).

To analyze inner retinal function, we examined the d-wave component of the ERG (Fig. 6A, arrowheads), which is a measure of the OFF-cone bipolar cell response [30, 31]. At PN 1-mo, there was no change, but by PN 3-mo, there was a 44.7% reduction (*p* ≤ 0.05, Fig. 6C) of d-wave amplitude *vs*. WT. By PN 18-mo, the d-wave was further reduced (by 56.5%, *p* ≤ 0.0001) in K42E mice compared to WT.

### Optokinetic reflex (OKR) in WT and K42E mice

**Fig. 7:**
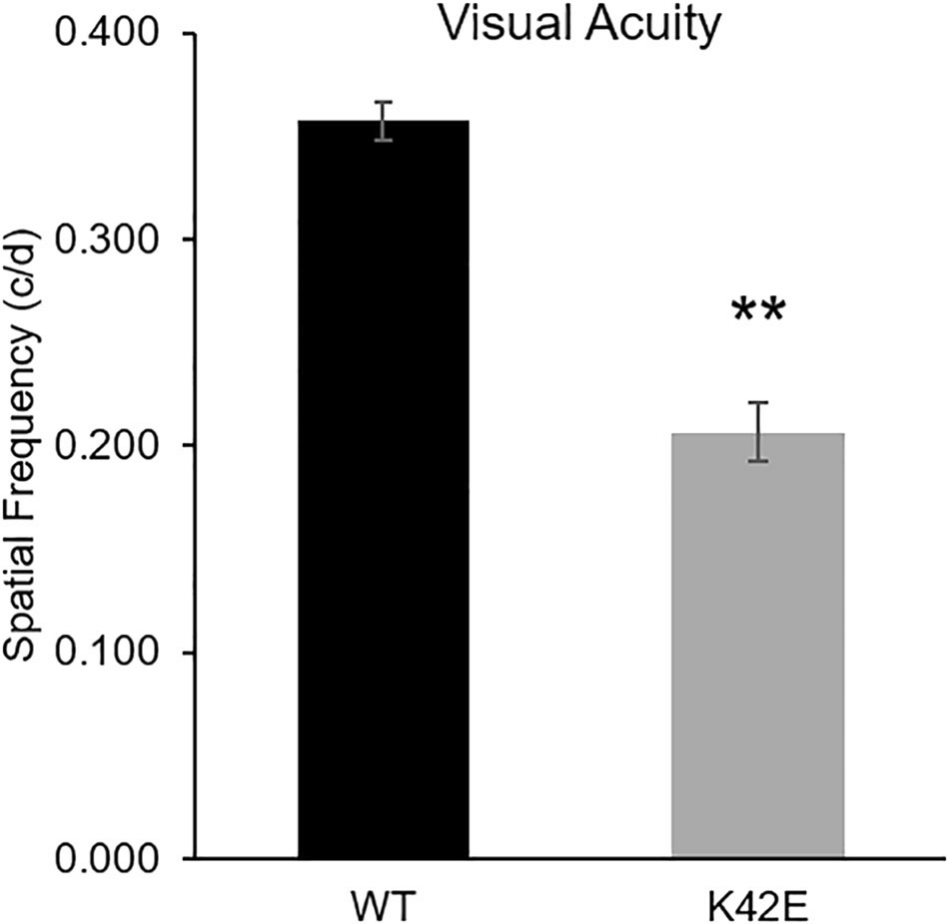
Visual acuity is decreased in K42E mice, relative to WT mice. The highest spatial frequency was determined (by OKR) of K42E (*gray*) and WT (*black*) mice at PN 12-mo. Visual acuity values for each mouse were averaged and demonstrate a reduction in K42E *vs*. WT mice. Statistical significance: **p ≤ 0.01. c/d, cycles/degree. K42E *n* = 10, WT *n* = 6.

As a measure of retina to brain transmission (i.e., the intactness of the visual pathway), we evaluated visual acuity by comparing optokinetic reflex (OKR) in WT and K42E mice. As shown in Fig. 7, we found a significant (42.3%, *p* ≤ 0.01) reduction in the response of K42E vs. WT mice. Hence, this further demonstrates that the K42E *Dhdd*s variant negatively impacts visual function in mice.

## Discussion

The lack of profound photoreceptor degeneration in the *Dhdds* K42E KI mouse model of RP59 [21] necessitated a deeper assessment of possible pathology. We used electrophysiological, molecular, and structural approaches to analyze the retina of K42E KI mice and found significant changes that define a novel phenotype. Furthermore, this phenotype has several similarities to the human disease, making the K42E KI mouse a valid and useful model of human RP59.

The non-syndromic phenotype is perplexing in that the protein encoded by the DHDDS gene is one subunit of the enzyme, CPT, which is ubiquitously required for several types of glycosylation in all cells. Absence of CPT results in embryonic lethality and most variants in DHDDS or the gene encoding NgBR, the other subunit of CPT, cause systemic disease [20]. We first tested the most likely hypothesis that RP59 is a CDG that somehow uniquely affects only the retina, as suggested by the groups who first reported RP59 [12, 13]. Having ruled out global changes in the most prevalent form of glycosylation, *N*-glycosylation [32], and not observing profound retinal degeneration as observed in patient studies [26, 33], we next examined alterations in Dol isoprenylogues, predicting a unique composition of Dol species in the mutant mouse retina. We found that similar statistically significant changes in the Dol profile were also observed in retina, liver, and brain tissue: Dol-17/Dol-18 ratio is dominant in mutant mice while Dol-18/Dol-19 ratio is dominant in WT mice (Fig. 1, Suppl. Fig. 1). While we cannot rule out that the shared altered profile somehow leads to a retina-specific effect, this seems unlikely. Furthermore, to date, there is little evidence from RP59 patients to indicate the disease should be classified a CDG. What has been reported in RP59 patients is a shift to shorter isoprenylogues and an increase in overall Dol content in plasma and/or urine [11, 26]. Even though the global change in Dol profile is less likely to cause disease, it does establish that the K42E change in mice has a parallel effect on Dol profiles as that reported in patients with DHDDS variants and in other related disorders [34].

We hypothesized that RP59 may be due to ectopic interactions of a conformationally modified CPT subunit or entire enzyme. This mechanism would require a previously unidentified interaction with a retina-specific component, such as what is frequently observed with dominant “gain-of-function” variants (e.g., most variants in rhodopsin) [35]. Consideration of DHDDS RP59 variants is further complicated by the fact that the disease is autosomal recessive, precluding a straightforward “gain-of-function” (dominant) mechanism. However, “gain-of-function” is supported by the recent finding of a specific deletion of the K42 codon in a DHDDS patient [19] presenting with only brain anomalies (cognitive impairment, abnormal motor control and epilepsy). Indeed, the authors speculate that this appears to be a DHDDS gain of function where an altered DHDDS conformation leads to a retina-restricted deleterious effect (e.g. “loss-of-function”). Cellular specialization is facilitated by restricted expression of unique genes or isoforms [36]. Possibilities include the mutant DHDDS subunit or the mutant enzyme ectopically interacting with some unknown retina-specific protein. Such an ectopic interaction could occur in the ER, where CPT normally functions. However, we cannot rule out that the CPT is mislocalized or abnormally elevated in some other compartment of retinal cells. Indeed, Dol has been found in several cellular compartments and has been suggested to have roles in membrane dynamics and in providing antioxidative properties [37, 38]. Thus, we cannot rule out that altered Dol levels contribute to the disease state through mechanisms unrelated to glycosylation. The relationship between Dol chain length and protein glycosylation efficiency remains to be resolved. Importantly, our results demonstrate that the K42E variant does not result in dramatic “loss-of-function” of DHDDS as elevated levels of Dol are found in all tissues examined in the KI mouse (Fig. 1, Suppl. Fig. 1). The elevation of dolichol levels in this RP59 model is a novel finding that has not been examined in RP59 patients.

Our previous finding of gliosis in the K42E KI mice indicated altered retinal function [21]. To identify potential regions of functional compromise in the retina, we assessed global changes in transcription. In addition to changes in genes associated with gliosis, such as GFAP [21], we observed significant changes in expression of genes involved in synaptogenesis and synaptic function (Suppl. Table 2), leading us to reexamine the synaptic regions of the retina. Inspection of OCT results revealed thinning of the INL (Fig. 2) and reduction in TRT consistent with inner retinal degeneration. Similarly, histologic analyses showed loss of INL cells and reduction in TRT (Fig. 3), leading us to examine the functional properties of the retina. Consistent with the morphologic change, a reduction in the ERG b-wave amplitude was seen, without attenuation of the a-wave response. Abnormalities in ERG, such as reduced b-waves with normal a-waves (termed negative ERG) have been linked to pre- or post-synaptic defects [27, 39]. These results are consistent with an inner retina defect likely involving photoreceptor to bipolar cell synaptic transmission, however, the specific cell types affected or the specific basis for the altered ERG, remain uncertain. To further dissect the functional pathways affected, we analyzed the ERG d-wave, a measure of the OFF-cone bipolar pathway response [40]. The marked reduction in d-wave amplitude implicates the bipolar OFF response in the diminished b-wave (Fig. 6B, D). Single cell analyses will be required to further dissect the functional loss in the K42E mouse retina.

The c-wave amplitudes of K42E mice are also reduced compared to control mice (Fig. 6A, C). Because the c-wave is a combined signal of hyperpolarized RPE and Müller cells [29, 30, 41], the source of the impairment remains unclear. With the reduced b/a-wave ratios and reduced d-wave amplitudes, a reduction in the c-wave is consistent with reduced potassium flow upon light stimulation [42, 43].

Implicit time has recently become an important aspect of ERG analysis to evaluate retinitis pigmentosa [44]. Implicit time analysis in our KI mouse model showed delayed b-wave in K42E mutant compared to WT mice under DA conditions (Fig. 5E, F), indicating longer bipolar cell recovery time, predicting slower flicker response or lower flicker fusion frequency.

In performing the optokinetic response test on WT and mutant mice, we were able to evaluate the signal transfer to the brain [45]. The visual acuity of the KI mice was significantly lower than that of WT mice, indicating a deficit in signal transmission (Fig. 7). Together with the ERG data, these findings could be explained by reduced synaptic transmission in the inner retina.

Previous studies showing markedly increased GFAP labeling [21] prompted us to perform TUNEL staining. TUNEL staining in mutant retina showed apoptosis in photoreceptor cells, Müller glia, horizontal cells, and bipolar cells (Fig. 4) at early ages, even before PN 2-mo. This indicates an early onset of disease pathology in the mutant mice, consistent with early detection of retinal degeneration in RP59 patients [26, 33, 46]. In addition, immunolabeling for VAMP2, PKC-α (Fig. 4A–C) and SYT-1 (Suppl. Fig. 2) indicated the presence of pathological remodeling of circuits associated with degenerative retinal disease [22, 23].

In summary, the *Dhdds* K42E KI mutant mouse exhibits retinal degeneration, most readily evident in the inner retina, which leads to an attenuated retinal response that involves compromised photoreceptor to inner retina synaptic transmission as well as attenuation of the RPE/Müller cell c-wave response. Several characteristic features observed in K42E mice are also found in RP59 patients: retinal degeneration, altered and progressively reduced DA and LA ERG amplitudes, increased ERG implicit times, and in both humans and mice, the pathology is non-syndromic. Hence, although no gross photoreceptor degeneration is evident in the mouse mutant, the K42E mouse is a valid model of RP59, which may have utility for elucidating the disease mechanism as well as to develop and test therapeutic interventions to retard or halt the progression of vision loss in RP59 patients.

## Materials and methods

### Animals

Generation and initial characterization of K42E KI mice were as described [21]. Homozygous K42E mice were compared to age-matched C57Bl/6J wild type (WT) mice. All animals were maintained on a standard 12/12 h light/dark cycle (20–40 lux ambient room illumination), fed standard rodent chow, provided water ad libitum, and housed in plastic cages with standard rodent bedding.

### Analysis of isoprenoid lipids

Mouse K42E and WT brain and liver specimens (0.2–0.4 g) were subjected to saponification (95 °C for 1 h in 3 ml of 25% (w/v) KOH in 65% (v/v) ethanol, *aq*)., and the nonsaponifiable lipids (including Dol and polyprenols) were then extracted three times with hexane. Pooled extracts were evaporated under a stream of nitrogen or argon, dissolved in 2-propanol and analyzed by HPLC/UV as described previously [47]. Retinas were extracted as previously described [48] and analyzed by LC/MS. Dol-13 was used as an internal standard.

### LC-MS analysis

Analysis of polyisoprenoids was performed using a Waters Acquity Ultra Performance Liquid Chromatography (UPLC) coupled with Waters TQ-MS triple-quadrupole mass spectrometer. The chromatographic separation of polyisoprenoids was carried out using reversed phase Accucore^TM^ C30 (150 mm × 2.1 mm) 2.6-µm HPLC column (Thermo Fisher Scientific) held at 60 °C. Mobile phase A consisted of 10 mM ammonium formate with 0.1% formic acid in acetonitrile/methanol/water (40:20:20, v/v/v) and mobile phase B of 10 mM ammonium formate with 0.1% formic acid in 2-pronanol/ acetonitrile (90:10, v/v). The flow rate was maintained at 0.6 mL/min throughout the whole analysis with an injection of 10 µL of sample using partial loop with needle overfill mode (PLNO). The gradient started with 10% of phase B ramped to 65% over 3 min. Next, steps of isocratic elution were performed with increasing percentage of phase B in quick, 5 min rampages: 8–13 min with 70% B; 13–18 min with 75% B; 18–23 min with 80% B; 23–28 min with 85% B; 28–33 min with 90% B; 33–38 min 92% B and 38–42 min with 99% B. Column equilibration was performed by returning to 10% B for 5 min. The total run time was 47 min.

The mass spectrometer operated in a selected-reaction monitoring mode (SRM). All mass spectra were collected using positive electrospray ionization (ESI) for ammonium adducts of polyisoprenoids [M + NH_4_]^+^. The tune method was optimized for all analytes with parameters as given: capillary voltage 2.0 kV, cone voltage 25.0 V, desolvation temperature 550 °C, desolvation gas flow 700 L/hour and cone gas flow 150 L/h. Data was acquired using MassLynx 4.1 (Waters) software and raw files were processed with TargetLynx XS 4.1 (Waters) software.

### Retinal transcriptome analysis

Total RNA was isolated from the retinas (2/sample) from PN 3-mo old WT and K42E mice (*n* = 3/genotype). RNA was analyzed using the NanoString® platform by the UAB Nanostring Core Laboratory (see https://nanostring.com/research-focus/neuroscience/). Data were combined and analyzed using NanoString® software nSolver 4.0 and Advanced Analysis 2.0, setting a fold-change cutoff of 2.0. To assess retinal networks, further analysis of the data was performed with Qiagen, Germantown, MD software, Ingenuity Pathway Analysis [49].

### Spectral-domain optical coherence tomography (SD-OCT)

Pupils were dilated and mice were anesthetized using ketamine-xylazine (50–5 mg/kg) as previously described [28]. In vivo imaging was performed on a Bioptigen Model 840 Envisu Class-R high-resolution SD-OCT instrument (Bioptigen/Leica, Inc; Durham, NC, USA). Retinal layer thicknesses were determined manually using Bioptigen InVivoVue® and Bioptigen Diver® V. 3.4.4 software. Data were analyzed and presented using Microsoft Excel software.

### Light microscopy

Mouse eyes were enucleated following euthanasia with 5% isoflurane and cervical dislocation, as previously described [50]. Light microscopy sections were cut at 0.8-μm thickness and stained with 0.1% Toluidine blue. Images were collected using an Olympus VS-120 microscope (BX61VS platform) running VS-ASW-2.9 software.

### Immunofluorescence labeling

Mice were euthanized and eyes harvested, fixed in 4% paraformaldehyde in PBS, and immunostained as previously described for cryosection [21]. Immunofluorescence was performed on cryosections from WT and K42E retinal tissues. Antibody information can be found in Table 1. TUNEL assay was performed as per manufacturer instruction (ABP Biosciences, A052), to detect apoptotic cells; DNase1 treated sections served as positive control. Tissues were visualized using a Zeiss Axioplan 2 wide-field epifluorescence microscope, and images were processed using FIJI-Image J and Huygens imaging software.

### Electroretinography (ERG)

Mice were dark adapted overnight, anesthetized with ketamine-xylazine (50 mg/kg–5 mg/kg), and corneas were anesthetized and dilated. ERGs were recorded with a contact lens electrode and 2.5% methylcellulose. A gold wire reference electrode was placed on the unstimulated eye. ERG experiments were done using a custom-built ERG system with fiber optics to deliver stimuli from a 100-Watt halogen bulb, or a high intensity LED light source. DA responses were recorded to brief flashes with intensity steps from 0.622 log photons/µm^2^ to 6.955 log photons/µm^2^. Following 3-min of rod saturation light adaptation, LA responses were recorded to flashes presenting at 6.955 log photons/µm^2^. The a-wave amplitudes were measured from baseline to the trough of the a-wave; b-wave amplitudes were measured from the trough of the a-wave to the peak of the b-wave. Implicit times were recorded in response to stimuli at the highest light intensities.

To measure the off-response (d-wave), a 5-s step of light was presented under DC recording; this stimulus also produced the c-wave, which is contributed by the retinal pigment epithelium (RPE) and Müller cells (2.27 log photons/µm^2^/sec) [29, 41, 51]. Amplitudes of the c-wave were measured from baseline to the peak of the c-wave and the d-wave (OFF-cone bipolar cell responses) were measured from the off transient.

All ERG responses were recorded and averaged using Lab View (version 6.4.1, National Instruments); and further analyses were performed Igor Pro Software (versions 8, 9).

### Optokinetic reflex (OKR)

To obtain an objective and quantitative assessment of visual function capacity, we employed the optokinetic reflex (OKR) method, a computer-based means of assessing visual [52] (OptoMotry^©^ 1.7.7; Cerebral Mechanics). Experiments were carried out as previously described in Gil et al. [53]. Spatial frequency was increased gradually, and visual acuity was determined when the mouse no longer reacted to the rotating gratings.

### Statistics

For all data, ANOVA and two-way Student’s *t*-test was used to determine statistical significance. Analysis of the ratio of isoprenylogue species was done using Wilcoxon-Mann-Whitney test (PROC NPAR1WAY, SAS software, version 9.4). Outliers were calculated and excluded from statistical analyses. Significance is indicated by *p ≤ 0.05, **p ≤ 0.01, and ***p ≤ 0.001.

## Supplementary information

CDD Checklist

Supplementary Material Legends

Shorter Dol chain lengths and higher total Dol -17/Dol-18 and Dol-18/Dol-19 ratios are found in K42E relative to WT mouse tissues.

SYT-1 staining indicates photoreceptor terminal retraction and impending cell death in the K42E retina.

Dol chain length percentages in retina, brain, and liver.

Altered synaptogenesis signaling pathway genes in K42E retina.

DA and LA ERG response averages.

## Data Availability

All data generated or analyzed during this study will be made available by the corresponding author upon reasonable request.
