# Supplementary material for: A Dhdds K42E knock-in RP59 mouse model shows inner retina pathology and defective synaptic transmission
Source: Cell Death Dis. 2023 Jul 13;14(7):420. doi: 10.1038/s41419-023-05936-4 (PMC10345138; doi:10.1038/s41419-023-05936-4)
Supplement: Supplementary file 2 — Supplementary Material Legends [file 41419_2023_5936_MOESM2_ESM.docx]

**Supplemental Figure 1. Shorter Dol chain lengths and higher total Dol -17/Dol-18 and Dol-18/Dol-19 ratios are found in K42E relative to WT mouse tissues.** Dol-15 to Dol-22 were analyzed by UPLC-MS in tissues from K42E and WT mice. Tissue analysis of Dol chain length percentages in brain **(A)** and liver **(B)** showed the WT dominant species to be Dol-18 and Dol-19. Analyses in K42E tissues showed the dominant species to be Dol-17 and Dol-18. Both Dol-17/Dol-18 **(C)** and Dol-18/Dol-19 **(D)** ratios were significantly higher in K42E brain and liver tissues compared to WT ratios. Statistical significance: ****p≤*0.001. K42E liver n=8, brain n=8; WT liver n=7, brain n=7.

**Supplemental Figure 2. SYT-1 staining indicates photoreceptor terminal retraction and impending cell death in the K42E retina.** Representative immunofluorescence images are presented with antibodies against synaptotagmin-1 (SYT-1, *magenta*) from retinal frozen sections of WT **(A)** and K42E **(B)** at PN 6-mo of age. Ectopic localization (*white arrows*) of SYT-1 was observed in the ONL and INL of the K42E retina. Abbreviations: ONL, outer nuclear layer; OPL, outer plexiform layer; INL, inner nuclear layer; IPL, inner plexiform layer; GCL, ganglion cell layer. Scale bar: 50 µm, both panels.

**Supplemental Table 1. Dol chain length percentages in retina, brain, and liver.** Dolichol content levels in tissues from WT and K42E retinas, brains, and livers relative to WT Dol-18 levels. This data was used to create Fig. 1 and Suppl. Fig. 1. ND= not done.

**Supplemental Table 2. Altered synaptogenesis signaling pathway genes in K42E retina.** From mRNA transcriptome analysis, up-regulated and down-regulated synaptogenesis genes are listed in respect to synaptic region – pre-synaptic, synaptic cleft, or post-synaptic – with a significance cutoff of 1.3.

**Supplemental Table 3. Dark- and Light-adapted ERG responses.** Shown are the data obtained for a- and b-wave, and b/a ratios used to create Fig. 5.
