# Supplementary figures and images for: A Dhdds K42E knock-in RP59 mouse model shows inner retina pathology and defective synaptic transmission
Source: Cell Death Dis. 2023 Jul 13;14(7):420. doi: 10.1038/s41419-023-05936-4 (PMC10345138; doi:10.1038/s41419-023-05936-4)

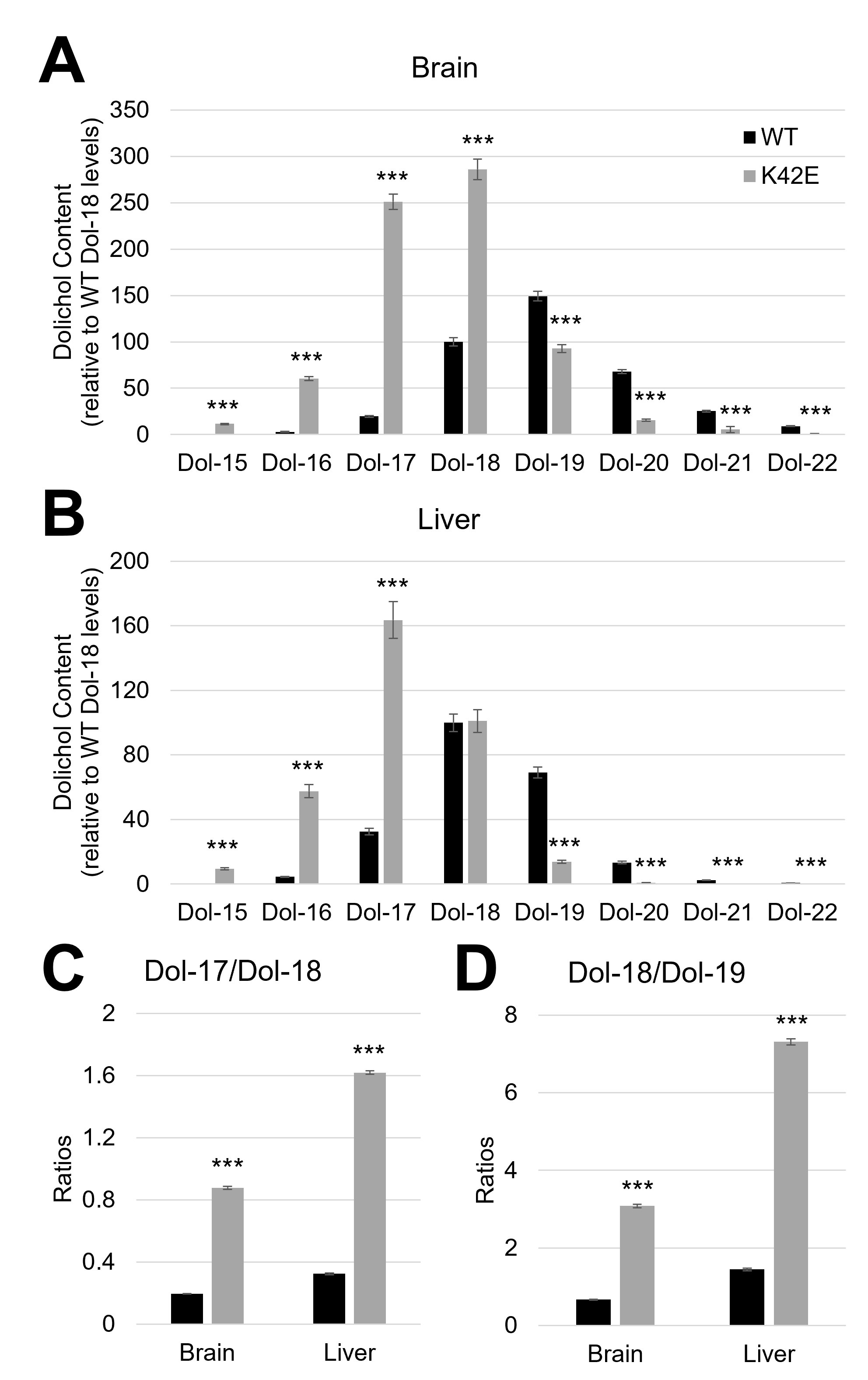

Supplement: Supplementary file 3 — Shorter Dol chain lengths and higher total Dol -17/Dol-18 and Dol-18/Dol-19 ratios are found in K42E relative to WT mouse tissues. [file 41419_2023_5936_MOESM3_ESM.tif]

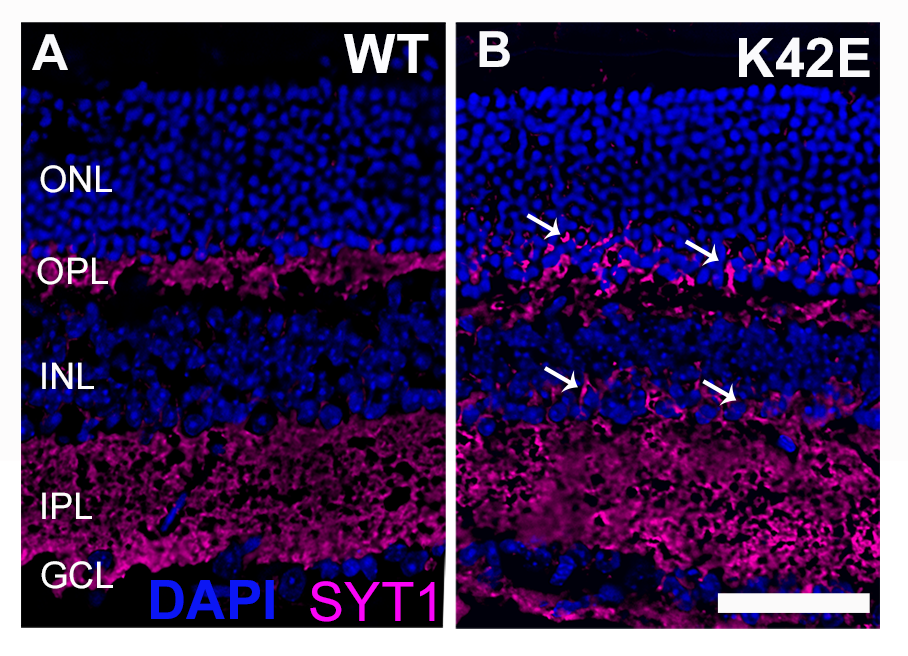

Supplement: Supplementary file 4 — SYT-1 staining indicates photoreceptor terminal retraction and impending cell death in the K42E retina. [file 41419_2023_5936_MOESM4_ESM.tif]
