# Supplementary material for: A Dhdds K42E knock-in RP59 mouse model shows inner retina pathology and defective synaptic transmission
Source: Cell Death Dis. 2023 Jul 13;14(7):420. doi: 10.1038/s41419-023-05936-4 (PMC10345138; doi:10.1038/s41419-023-05936-4)
Supplement: Supplementary file 5 — Dol chain length percentages in retina, brain, and liver. [file 41419_2023_5936_MOESM5_ESM.docx]

| **Retina** | | | | | | | | |
| --- | --- | --- | --- | --- | --- | --- | --- | --- |
|  | **Dol-15** | **Dol-16** | **Dol-17** | **Dol-18** | **Dol-19** | **Dol-20** | **Dol-21** | **Dol-22** |
| WT | 4±3 | 2±1 | 29±1 | 100±3 | 66±5 | 14±4 | 8±5 | ND |
| K42E | 6±5 | 64±12 | 203±12 | 131±9 | 14±2 | 0±0 | 26±19 | ND |
| **Brain** | | | | | | | | |
|  | **Dol-15** | **Dol-16** | **Dol-17** | **Dol-18** | **Dol-19** | **Dol-20** | **Dol-21** | **Dol-22** |
| WT | 0±0 | 3±0 | 20±1 | 100±4 | 149±5 | 68±2 | 25±1 | 9±0 |
| K42E | 12±1 | 61±2 | 251±8 | 286±11 | 93±4 | 15±1 | 5±3 | 1±1 |
| **Liver** | | | | | | | | |
|  | **Dol-15** | **Dol-16** | **Dol-17** | **Dol-18** | **Dol-19** | **Dol-20** | **Dol-21** | **Dol-22** |
| WT | 0±0 | 4±0 | 32±2 | 100±5 | 69±4 | 13±1 | 2±0 | 1±0 |
| K42E | 10±1 | 58±4 | 164±11 | 101±7 | 14±1 | 1±0 | 0±0 | 0±0 |
