# Supplementary material for: A Dhdds K42E knock-in RP59 mouse model shows inner retina pathology and defective synaptic transmission
Source: Cell Death Dis. 2023 Jul 13;14(7):420. doi: 10.1038/s41419-023-05936-4 (PMC10345138; doi:10.1038/s41419-023-05936-4)
Supplement: Supplementary file 6 — Altered synaptogenesis signaling pathway genes in K42E retina. [file 41419_2023_5936_MOESM6_ESM.docx]

| Synaptic region | Up-regulated | | Down-Regulated |
| --- | --- | --- | --- |
| Pre-synaptic  **18** Up-regulated  **2** Down-regulated | ADCV  AP-2  Camk2  Cdh1  DNAJC-5  Epha/b  Nrxn1  NSF  N-VDCC | PKA  RAB3A  Snap25  Snca  Stx1a  Syn1  Syt1  Unc13a | Cplx-1  Munc18 |
|  | | | |
| Synaptic cleft | ApoE | | Bdnf |
|  | | | |
| Post-synaptic  **35** Up-regulated  **11** Down-regulated | Adcy  Akt  Ampar  Arp2/3  Bad  Braf  Cam  Cdk5  Chn1  Crk  Crkl  Ctnnb1  Dlg4  Epha  Grb2  Iraf1  Itpri | Lrp1  Mapt  Mglur  Mrp2-b  Nmda  Pak1  Pka  Pkcd  Prkce  Rac1  RasGrp1  Rhoa  Sfk  Sho  Syngap  Tiam1  Wasf1 | Creb  Erk1/2  Grb2  Gsk3b  Iraf1  Mtor  p38Mapk  Pi3k  Ras  Rasgrf1 |
