# Supplementary material for: A Dhdds K42E knock-in RP59 mouse model shows inner retina pathology and defective synaptic transmission
Source: Cell Death Dis. 2023 Jul 13;14(7):420. doi: 10.1038/s41419-023-05936-4 (PMC10345138; doi:10.1038/s41419-023-05936-4)
Supplement: Supplementary file 7 — DA and LA ERG response averages. [file 41419_2023_5936_MOESM7_ESM.docx]

**Dark-adapted ERG Responses**

|  | **WT** | | | **K42E** | | | |
| --- | --- | --- | --- | --- | --- | --- | --- |
| **Age (mo)** | **a-wave** | **b-wave** | **b/a ratio** | **a-wave** | **b-wave** | **b/a ratio** |  |
| **1** | 241±32 | 688±101 | 2.9±0.1 | 222±19 | 526±45 | 2.4±0.1 |  |
| **2** | 200±67 | 542±181 | 2.8±0.1 | 262±30 | 416±50 | 1.3±0.04 |  |
| **3** | 296±54 | 775±163 | 2.8±0.1 | 323±11 | 422±27 | 1.3±0.1 |  |
| **6** | 280±59 | 785±172 | 2.6±0.1 | 412±62 | 484±68 | 1.1±0.03 |  |
| **8** | 229±19 | 528±34 | 2.2±0.1 | 434±92 | 370±89 | 0.9±0.1 |  |
| **9** | 218±33 | 548±69 | 2.0±0.1 | 262±34 | 341±100 | 0.7±0.1 |  |
| **12** | 208±38 | 438±77 | 2.4±0.1 | 184±23 | 146±21 | 0.7±0.1 |  |
| **18** | 129±21 | 312±37 | 2.4±0.1 | 139±22 | 100±18 | 0.7±0.04 |  |

**Light-adapted ERG Responses**

|  | **WT** | | | **K42E** | | |
| --- | --- | --- | --- | --- | --- | --- |
| **Age (mo)** | **a-wave** | **b-wave** | **b/a ratio** | **a-wave** | **b-wave** | **b/a ratio** |
| **1** | 156±16 | 318±48 | 2.2±0.1 | 177±14 | 273±26 | 1.5±0.1 |
| **2** | 119±49 | 246±87 | 2.3±0.2 | 140±23 | 138±14 | 0.8±0.3 |
| **3** | 147±18 | 341±42 | 2.5±0.1 | 178±13 | 152±11 | 0.8±0.1 |
| **6** | 86±13 | 216±41 | 2.3±0.03 | 254±86 | 220±65 | 0.9±0.03 |
| **8** | 106±15 | 290±30 | 2.3±0.2 | 226±31 | 166±20 | 0.8±0.1 |
| **9** | 120±6 | 290±22 | 2.1±0.01 | 142±22 | 183±83 | 0.5±0.02 |
| **12** | 107±18 | 230±40 | 2.2±0.2 | 151±16 | 73±12 | 0.5±0.1 |
| **18** | 86±10 | 169±16 | 2.0±0.1 | 111±14 | 62±10 | 0.6±0.2 |
